# Asthma Is Inversely Associated with *Helicobacter pylori* Status in an Urban Population

**DOI:** 10.1371/journal.pone.0004060

**Published:** 2008-12-29

**Authors:** Joan Reibman, Michael Marmor, Joshua Filner, Maria-Elena Fernandez-Beros, Linda Rogers, Guillermo I. Perez-Perez, Martin J. Blaser

**Affiliations:** 1 Department of Medicine, New York University School of Medicine, New York City, New York, United States of America; 2 Department of Environmental Medicine, New York University School of Medicine, New York City, New York, United States of America; Yale University School of Medicine, United States of America

## Abstract

**Background:**

Microbial exposures have been suggested to confer protection from allergic disorders and reduced exposures to gastrointestinal microbiota have been proposed as an explanation for the increase in asthma prevalence. Since the general prevalence of *Helicobacter pylori* has been decreasing, we hypothesized that *H. pylori* serostatus would be inversely related to the presence of asthma.

**Methods:**

Adults were recruited to participate in the New York University (NYU)/Bellevue Asthma Registry in New York City. Adult asthma cases (N = 318) and controls (N = 208) were identified and serum IgG antibodies to *H. pylori* whole cell antigens or the immunodominant CagA antigen were measured.

**Results:**

As expected, the asthma cases and controls differed with respect to atopy and lung function. Seropositivity to *H. pylori* or CagA antigen was present in 47.1% of the total case and control study population. Asthma was inversely associated with CagA seropositivity (OR = 0.57, 95% CI = 0.36–0.89). Median age of onset of asthma (doctor's diagnosis) was older (21 years) among individuals with CagA*+* strains than among *H. pylori-* individuals (11 years) (*p* = 0.006).

**Conclusion:**

These data are consistent with the hypothesis that colonization with CagA+ *H. pylori* strains is inversely associated with asthma and is associated with an older age of asthma onset in an urban population. The data suggest *H. pylori* as a marker for protection.

**Trial Registration:**

ClinicalTrials.gov NCT00212537

## Introduction

The prevalence of atopy and asthma has increased worldwide [1]. The “hygiene hypothesis,” that reduced childhood exposure to microorganisms modifies polarized Th1/Th2 responses leading to more allergic disorders, has been proposed to explain this increase [2]. Relevant microbial exposures may include gastrointestinal biota. Intestinal microbiota differ between healthy infants in countries with low or high allergy prevalence, as well as between allergic and non-allergic infants [3]. Early exposure to orofecal microbes such as Hepatitis A, appears to protect against allergen sensitization [4]–[7], and in Italian military recruits and Danish adults, HAV, *Toxoplasma gondii*, and *Helicobacter pylori*, are inversely associated with atopy [8], [9].

*H. pylori*, gram-negative, microaerophilic gastric bacteria persistently colonize much of the world's population. Whereas nearly all adults are *H. pylori*-positive in developing countries, with socioeconomic development, prevalence has decreased substantially [10], [11]. *H. pylori* is almost exclusively acquired in childhood [12], [13] and antibody responses are present for decades or for life, consistent with the persistent gastric colonization [10], [11], [14]. *H. pylori* virulence is affected by the presence of the 35-40-kb *cag* pathogenicity island that can be detected by identification of the *cagA* gene or its product (CagA) [15]. CagA*+* strains are more host-interactive [15], [16]. *H. pylori* colonization induces continuous gastric inflammation, which is more pronounced with *cagA+* strains [17], and leads toward diminished gastric acidity [18]. Serologic assays to detect antibodies to the CagA protein enhance overall detection of *H. pylori*, and specifically detection of the more interactive (CagA+) organisms [19]. Antibodies to CagA persist for at least two decades in the absence of antimicrobial treatments that eliminate *H. pylori* [14].

We hypothesized that the presence of *H. pylori* antibodies would be inversely related to asthma and that *cagA+* strains of *H. pylori* would have a more pronounced inverse relationship with asthma. A recent cross-sectional study of adults in Iceland, Estonia and Sweden suggested an inverse association of antibodies to *H. pylori* and self-reported hay fever or asthma [20]. In the NHANES III population, we demonstrated an inverse association of ever having had asthma with a *cagA*+ *H. pylori* strain [21] and in the NHANES IV population, in which CagA testing was not done, we found inverse associations of *H. pylori* status with childhood-onset asthma and allergic disorders [22]. We now provide evidence showing an inverse association of CagA serology with asthma in a case control study of an additional well-characterized and separate adult urban population.

## Methods

### Study population

Asthma cases and non-asthma controls were recruited to participate in the New York University (NYU)/Bellevue Asthma Registry in New York City. The registry was approved by the Institutional Review Board of the New York University School of Medicine. All cases and controls signed informed consent. Letters informing patients of positive *H. pylori* serology were sent under an IRB-approved protocol. Cases were referred to the registry by the Bellevue Hospital Center Asthma Clinic and local clinics. Controls were referred by asthma cases and by enlisting individuals directly from the community and from other programs within Bellevue Hospital Center. A number of referrals from the cases were unintentionally genetically related and these individuals were accounted for in the statistical methods. Cases and controls were excluded if they were <18 or ≥65 years old; were current smokers; had a history of >10 pack-year tobacco use; or had an unstable cardiac disease, uncontrolled hypertension, lung disease other than asthma, or neuromuscular disease.

Questionnaires and evaluations were completed for 573 persons. Subjects were considered to have a diagnosis of “asthma” based on their response to questions derived from validated questionnaires [23], [24] used for international studies of asthma. All patients were seen by a physician or nurse with extensive experience in asthma diagnoses and management. Because most of these adult patients were using chronic medication for asthma or had longstanding disease, a 12% change in FEV_1_ was not used as a criterion for diagnosis. We confirmed our diagnosis with the published algorithm of Enright et al. [25]. Twelve persons who could not be classified as either having asthma or being asthma-free and 35 individuals without serum samples were excluded from statistical analyses. Race and ethnicity were self-classified. The final study population included 526 subjects (318 asthma cases and 208 controls).

### Serum antibody analysis

Serum anti-*H. pylori* IgG antibody levels were determined by ELISA using whole cell antigens [19]. CagA status was determined by a separate ELISA, based on the presence of serum IgG antibodies against orv220, a 65 kDa recombinant CagA truncated protein [19], [26]. Absence of *H. pylori* was defined as negativity in both the whole cell and CagA assays (*H. pylori* negative). Individuals were defined as colonized with *cagA* negativ*e H. pylori* strains if they had antibodies to the whole cell antigen but not to CagA (*H. pylori+*/CagA−). Subjects were defined as being colonized with *cagA+ H. pylori* strains if they were positive for CagA antibodies, whether or not they were positive for *H. pylori* antibodies (CagA*+*). Thirty one persons (5.9%) were CagA*+* but had *H. pylori* serological determinations that did not reach positive values, a finding consistent with previous studies in *H. pylori* culture-positive subjects [27].

### Allergy testing

Measurements of total serum IgE (total IgE) and allergen-specific IgE for allergens considered significant for the Northeastern United States were performed in a commercial laboratory (Pharmacia ImmunoCAP assay; Quest Diagnostics; Teterboro, NJ). Allergen results were available for 525 of the 526 subjects. An allergen-specific IgE level >0.35 kilo-international units (kIU)/L was considered positive.

### Spirometry

Pre- and post-bronchodilator spirometry was performed according to American Thoracic Society guidelines [28]; normal values were obtained from Hankinson et al. [29]. Values were obtained on 516 subjects, but were not available for 10 subjects.

### Statistical Methods

Non-parametric Wilcoxon and Kruskall-Wallis tests were used for crude comparisons of quantitative variables among groups, and the chi-squared test used for comparisons of categorical variables. In multivariable analyses, generalized estimating equations (GEE) were used to confirm the findings because of the presence of matched sets of individuals that occurred when asthma cases referred family members to the study. Data from genetically related cases and controls (n = 104) were entered as “repeated measures” in GEE logistic and linear regression analyses to account for the potentially correlated nature of observations among related individuals [30]. GEE was used with a logit link for logistic regression analyses of risk factors for asthma; risk factors for allergen-specific IgE, and separately, for seropositivity to either *cagA− or cagA*+ strains of *H. pylori*. In multivariable analyses, we first included all potential confounders in models and then dropped from the models those potential confounders that did not substantially affect the odds ratios of the variables of interest. GEE analyses were conducted using SAS 9.1 Proc GENMOD (SAS Institute Inc., Cary, NC, USA, 2002). Kaplan-Meier estimation and Cox proportional hazards regression were used to investigate correlates of age of diagnoses of asthma.

## Results

### Characteristics of the study groups

Characteristics of the asthma cases and non-asthma controls are shown in Table 1. Cases and controls were similar in age and gender. Cases were more often Hispanic, and income levels were lower in the cases than in the controls. Hispanic ethnicity was not associated with asthma status, once income and race were adjusted for via logistic regression. As expected, total IgE was elevated in the cases compared to the controls, and there was a significant association of asthma with atopy, as defined by the presence of at least one allergen-specific IgE. Lung function parameters including post-bronchodilator forced expiratory volume in one second (FEV_1_), post-bronchodilator forced vital capacity (FVC), and the ratio of FEV_1_/FVC were reduced in the cases compared to controls. These characteristics are consistent with expectations for an asthma population compared to a control population.

**Table 1:** Characteristics of the case control study population.

Characteristic	Asthma cases (N = 318)	Controls (N = 208)	Crude Odds ratio (95% CI)	Adjusted* Odds ratio (95% CI)	*p*-value†
**Age–year** (median, IQR)	34 (18–64)	38 (18–64)			0.4
**Sex - no. (%)**					
Male	95 (29.9)	69 (33.2)	1.0		
Female	223 (70.1)	139 (66.8)	1.17 (0.8–1.7)	0.97 (0.7–1.4)	
**Race–no. (%)**					
White	239 (75.2)	123 (59.1)	1.0	1.0	
Black	53 (16.7)	40 (19.2)	0.68 (0.42–1.1)	0.70 (0.43–1.1)	
Asian and other	26 (8.2)	45 (21.6)	0.29 (0.17–0.52)	0.35 (0.20–0.61)	
**Hispanic ethnicity–no. (%)**					
No	130 (40.9)	131 (63.0)	1.0	1.0	
Yes	188 (59.1)	77 (37.0)	2.5 (1.7–3.6)	1.4 (0.8–2.2)	
**Yearly income–no. (%)**					
<15 K	132 (41.5)	40 (19.2)	1.0	1.0	
15–49 K	82 (25.8)	64 (30.8)	0.39 (0.23–0.65)	0.39 (0.24–0.63)	
50–99 K	43 (13.5)	56 (26.9)	0.23 (0.13–0.41)	0.26 (0.15–0.45)	
≥100 K	11 (3.5)	19 (9.1)	0.18 (0.07–0.43)	0.20 (0.09–0.47)	
No response or refused	50 (15.7)	29 (13.9)	0.52 (0.28–0.97)	0.54 (0.30–0.98)	
**Atopic status** a **- no. (%)**					
Non-atopic	74 (23.3)	95 (45.9)	1.0		
Atopic	244 (76.7)	112 (54.1)	2.80 (1.9–4.2)	3.4 (2.2–5.0)	
**Total IgE - U/ml** (median, IQR)	129 (40–386)	42 (15–140)			<0.0001†
**Spirometry - % predicted** (median, IQR)					
Post bd FEV_1_	86 (74–97)	92 (85–101)			<0.0001
Post bd FVC	88 (78–98)	91 (82–100)			<0.02
Post bd FEV_1_/FVC	81 (75–85)	85 (81–87)			<0.0001

† *p*-values are from the Wilcoxon test and are provided for quantitative variables.

* Adjusted for income (using 5 categories shown in table) and race via logistic regression.

a Atopy defined as presence of any allergen-specific IgE at a level >35 kIU/L.

### *H. pylori* status and asthma

Table 2 shows the crude and adjusted odds ratios (OR) for asthma and H. pylori serostatus. Although there was a suggestion of an association of asthma with CagA+ status, the crude OR for asthma associated with *H. pylori+/*CagA*−* or CagA+ status failed reach significance. In contrast, after adjustment for race and income, there was a significant inverse association of asthma and CagA+ status with an OR of 0.63 (95% CI = 0.41–0.98). We also examined the association of atopy and CagA+ status with asthma using GEE logistic regression analysis to adjust for age, race, Hispanic ethnicity, income, and the genetic relatedness among some of the subjects (Table 3). As expected, atopy was associated with asthma. The significant inverse association of CagA*+* status with asthma was again demonstrated (OR = 0.57, 95% CI = 0.36–0.89). Analyses that treated all subjects as independent also yielded similar results (data not shown). We also repeated the analyses limiting subjects to those who were neither genetically related nor referred by one another (N = 402). Unconditional logistic regression in this group, adjusted for age, income, education, race/ethnicity, and atopy continued to yield an inverse association of asthma with CagA+ status (OR = 0.49, 95% CI = 0.28–0.85). The inverse OR associated with CagA+ status also was not substantially altered when atopy was excluded from the model. Inclusion of variables representing presence of allergen-specific IgE antibodies or of log_10_IgE in the multiple logistic regression model for asthma also had no major impact on our primary finding of an inverse association with CagA*+ H. pylori* strains.

**Table 2:** Association between *H. pylori* serostatus and asthma in asthma cases (N = 318) and non-asthma controls (N = 208).

	Asthma cases N (%)	Controls N (%)	Crude OR (95% CI)	Adjusted* OR (95% CI)
***H. pylori*** ** status**				
*H. pylori−/*CagA−	171 (53.8)	108 (51.9)	1.0	1.0
*H. pylori+/*CagA−	68 (21.4)	35 (16.8)	1.23 (0.74–2.03)	0.94 (0.57–1.57)
CagA+	79 (24.8)	65 (31.3)	0.77 (0.50–1.18)	0.63 (0.41–0.98)

* Adjusted for income (using 5 categories shown in Table 1) and race (white, black, other) via logistic regression.

**Table 3:** Association between *H. pylori* status or atopy and asthma using generalized estimating equation (GEE) multiple logistic regression analysis (N = 525).

Risk factor	Value	OR^a^	95% CI	*p*-value
*H. pylori* status	*H. pylori*−	1.0	–	
	*H. pylori+/*CagA−	0.74	(0.41–1.3)	0.39
	CagA*+*	0.57	(0.36–0.89)	0.02
Atopy^b^	No	1.0	–	
	Yes	3.39	(2.20–5.20)	<0.0001

a Multivariate analysis performed using GEE and adjusted for age (in years), education (in years), income, race (white, black, other) and Hispanic ethnicity.

b Atopy defined as any allergen-specific IgE.

### *H. pylori* as an asthma modifier

We examined the relationship between *H.pylori* serostatus and IgE. Log_10_IgE was not associated with either *H. pylori+*/CagA− status or CagA*+* status after adjustment for differences in race, Hispanic ethnicity, and age (GEE, data not shown). No relationship was identified between atopy, and either *H. pylori*+/CagA− status or CagA+ status. Among atopic subjects (N = 356), 19.7% were *H. pylori*+/CagA− and 27.3% were CagA+, whereas among non-atopic subjects (N = 169), 19.3% were *H. pylori*+/CagA− and 27.8% were CagA+ (*p* = 0.99). Atopy also was not associated with *H. pylori* status in a multivariable GEE logistic regression model that included adjustments for race, Hispanic ethnicity, education and income. Similarly, atopy was not associated with *H. pylori* status in the control population (*p* = 0.8).

In addition, we examined whether *H. pylori* serostatus was an asthma modifier using post-bronchodilator FEV_1_ and FEV_1_/FVC as surrogates of asthma severity. There was a significant difference in FEV_1_ among the individuals with asthma who were CagA*+* (N = 76, median % predicted = 82.0 (interquartile range, IQR = 71–92) compared to those who were *H. pylori* negative (N = 170, median % predicted = 90.0, IQR = 77–100) (*p* = 0.008), although FEV_1_/FVC was not different between the two groups (median = 80.0% predicted, IQR = 74.0–86.5 in CagA+ subjects and 82.0%, IQR = 76–85 in *H. pylori*− subjects) (*p* = 0.4).

**Figure 1:**
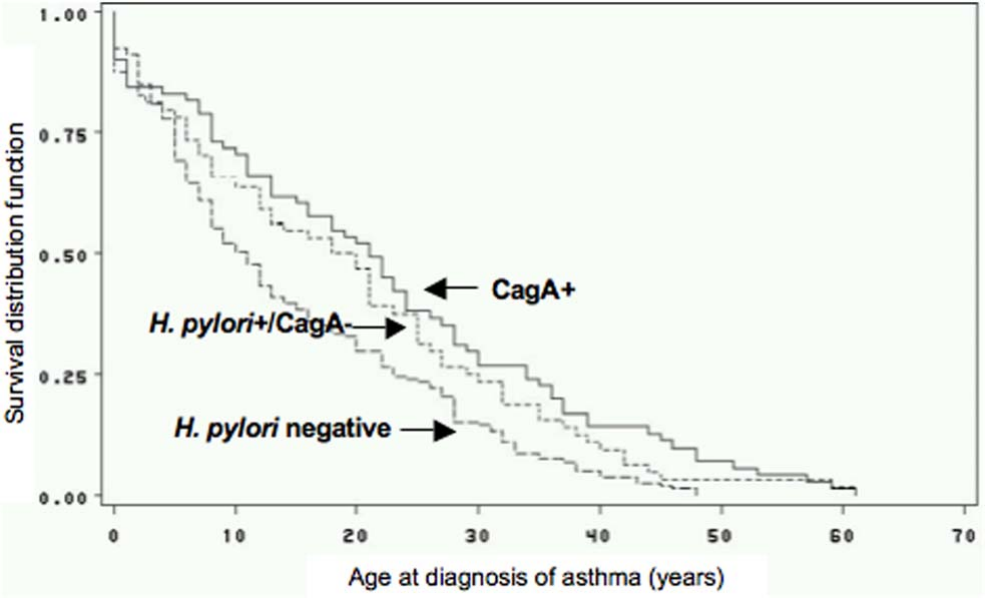
Kaplan-Meier estimation of asthma-free survival among 294 adults with asthma according to *H. pylori* status (– – – *H. pylori* negative (n = 159); ---- *H. pylori*+/CagA− (n = 64); CagA+ (n = 71).^a^ ^a^ (Data for age of asthma onset are not available for 24 of the 318 cases).

We next asked whether *H. pylori* serostatus was associated with the age of onset of asthma. Age of onset of asthma was similar among the *H. pylori+*/CagA− individuals (N = 64, median age = 19, IQR = 6–30) and the CagA+ individuals (N = 71, median age = 21, IQR = 8–34), but was substantially lower among the *H. pylori negative* individuals (N = 159, median age = 11 y, IQR = 5–23) (*p* = 0.006). Similar differences in age were noted when we assessed age at onset of symptoms (data not shown). We performed Kaplan-Meier analysis of the probability of age-related survival until a doctor's diagnosis of asthma (Figure 1) assuming that acquisition of *H. pylori* occurred close to the time of birth, as suggested in the literature [13], [17], [31], [32]. Cox regression analysis adjusted for age and income (adjustment for race/ethnicity did not affect the results of this analysis) suggested a reduced hazard ratio (HR) in CagA*+* individuals compared to *H. pylori−* individuals (HR = 0.74, 95% CI = 0.54–1.02) whereas the HR for *H. pylori+*/CagA− individuals compared to *H. pylori−* individuals was not substantially different from 1 (HR = 0.93, 95% CI = 0.67–1.29). We further modeled ages at doctor's diagnosis of asthma by GEE to take into account potentially correlated times of diagnosis among genetically related family members and to adjust for potential confounders. This analysis showed a significantly greater age at doctor's diagnosis of asthma among CagA+ subjects compared with *H. pylor*i-negative subjects (*p* = 0.02).

## Discussion

Reduced exposure to orofecal organisms has been suggested as an explanation for the increasing prevalence of atopy and asthma, but studies of multiple organisms have had conflicting results [5]. We studied a racially and ethnically diverse urban population in a case control study to examine the relationship between asthma and *H. pylori* serostatus. *H. pylori* seroprevalence was common in both cases and controls, in accordance with national estimates [33]. Our analysis identified a trend towards an inverse association between *H. pylori* and asthma that became significant when we examined individuals who carried cagA+ *H. pylori* strains. Our data provide evidence that in an urban population, asthma is inversely associated with serologic evidence of the presence of cagA+ H. *pylori* strains. We used several different analytical approaches, and analytical results were highly consistent. Our findings support and expand upon our recent cross-sectional study of separate populations of individuals with an asthma diagnosis in the NHANES III and NHANES IV populations [21], [22], and now include subjects with spirometry and serum IgE determinations. Our analysis of age of onset of disease in an adult population, in which we detect a delay on onset of asthma in CagA+ individuals, is consistent with the age relationships reported in the two NHANES populations.

We did not detect an effect of *H. pylori* serostatus on total IgE or the presence of atopy in our population. This finding suggests that the ability of *H. pylori* exposure to modify asthma may be mediated via mechanisms other than those associated with IgE levels. Interestingly, although we detected a delay in the onset of asthma associated with the presence of CagA+ serology, FEV_1_ was reduced in this group. The finding of reduced lung function in individuals with a later age of onset of asthma is consistent with those recently reported [34]–[36].

Potential mechanisms by which *H. pylori* could alter asthma presentation include immune modifications or an effect on gastro-esophageal reflux disease (GERD). Although *H. pylori* colonization recruits neutrophils, T and B lymphocytes and macrophages to the stomach [17], *H. pylori* generally persists for the host's lifetime [37], reflecting immune evasion and modification of host inflammatory, innate, and adaptive immune responses [10], [12], [38], [39]. *H. pylori* may alter the polarized Th1/Th2 T cell response through dendritic cell-mediated T-cell expression of IL-12, TNF-α, and IFN-γ [40]–[42], and *cagA* translocation induces IL-12 production [43]. *H. pylori* colonization induces regulatory T cells including CD4^+^ CD25^+^ T cells that express the forkhead box P3 transcription factor (Foxp3) [44], [45] and also induces indoleamine 2,3-dioxygenase, mechanisms that suppress T cell function [46]. IL-10 expression is increased in the gastric mucosa of children carrying *H. pylori* [47]. Such immunosuppressive and immunomodulatory effects have the potential to alter the effector phase of asthma as recently shown in murine models of helminth infection [48]. *Helicobacter pylori* upregulate Toll-like receptor 4 (TLR4) [49], and although results are complex, human and murine studies suggest that activation of TLR4 may be protective against allergic asthma [50].

Associations between GERD and asthma also are well-established [51]–[53]. Longitudinal studies show that asthma is a risk factor for development of GERD, and that GERD can trigger asthma [51]–[53]. *H. pylori*, especially *cagA+* strains, are inversely associated with GERD [12], [54]. Although we did not specifically assess for GERD in this study, the possibility exists that the inverse association between *H. pylori* and asthma reflects protection from GERD.

An alternative explanation for our findings is that *H. pylori* seronegativity is a surrogate for other phenomena such as the presence or absence of other indigenous biota, or merely reflects cumulative early life exposure to antibiotics, identified as a risk factor for asthma. Although possible, the specificity of the relationship to *cagA+* strains argues against this point. There are some potential limitations to this study. Although we do not know the age at which *H. pylori* was acquired in the cases or controls, multiple studies have demonstrated that nearly all acquisition that occurs does so at an early age, usually before the age of five [12], [13]. *H. pylori* antibodies reflect the present carriage of *H. pylori*, and its prior elimination due to antibiotic exposure could lead to seronegativity. Thus, the current serostatus could under-estimate *H. pylori* acquisition, but not persistence, since with long-term carriage of *H. pylori*, antibody levels are stable [12], [15].

One potential confounding factor could be greater antibiotic use in asthma cases than in controls, which would bias toward consequent elimination of *H. pylori*. The association of the inverse association with childhood-onset but not later-age onset asthma in this study (Figure 1), and two other recent studies [21], [22] as well as the specificity of the effect with *cagA^+^* positivity argues against that point. However, prospective studies will be needed to clarify this question.

Because adults were studied, the association of *H. pylori* with delay in asthma onset may be confounded by recall bias or delay in doctor diagnosis. Although these issues suggest the need for future prospective studies, our findings support those of our recent cross-sectional studies of NHANES populations [21], [22].

In conclusion, our data suggest that *H. pylori*, and specifically, CagA positivity is inversely associated with asthma and with a delay in the onset of asthma. That the association was strongest with *cagA+ H. pylori* strains suggests that the more intensive host-interaction of these organisms may influence disease expression.

## References

[1] Anderson HR (2005). Prevalence of asthma.. Bmj.

[2] Strachan DP (2000). Family size, infection and atopy: the first decade of the “hygiene hypothesis”.. Thorax.

[3] Sepp E, Julge K, Vasar M, Naaber P, Bjorksten B (1997). Intestinal microflora of Estonian and Swedish infants.. Acta Paediatr.

[4] Bjorksten B (2004). Effects of intestinal microflora and the environment on the development of asthma and allergy.. Springer Semin Immunopathol.

[5] Ramsey CD, Celedon JC (2005). The hygiene hypothesis and asthma.. Curr Opin Pulm Med.

[6] Matricardi PM, Rosmini F, Ferrigno L, Nisini R, Rapicetta M (1997). Cross sectional retrospective study of prevalence of atopy among Italian military students with antibodies against hepatitis A virus.. Bmj.

[7] Matricardi PM, Rosmini F, Panetta V, Ferrigno L, Bonini S (2002). Hay fever and asthma in relation to markers of infection in the United States.. J Allergy Clin Immunol.

[8] Matricardi PM, Rosmini F, Riondino S, Fortini M, Ferrigno L (2000). Exposure to foodborne and orofecal microbes versus airborne viruses in relation to atopy and allergic asthma: epidemiological study.. Bmj.

[9] Linneberg A, Ostergaard C, Tvede M, Andersen LP, Nielsen NH (2003). IgG antibodies against microorganisms and atopic disease in Danish adults: the Copenhagen Allergy Study.. J Allergy Clin Immunol.

[10] Dooley CP, Cohen H, Fitzgibbons PL, Bauer M, Appleman MD (1989). Prevalence of Helicobacter pylori infection and histologic gastritis in asymptomatic persons.. N Engl J Med.

[11] Kosunen TU, Aromaa A, Knekt P, Salomaa A, Rautelin H (1997). Helicobacter antibodies in 1973 and 1994 in the adult population of Vammala, Finland.. Epidemiol Infect.

[12] Blaser MJ, Atherton JC (2004). Helicobacter pylori persistence: biology and disease.. J Clin Invest.

[13] Rowland M, Daly L, Vaughan M, Higgins A, Bourke B (2006). Age-specific incidence of Helicobacter pylori.. Gastroenterology.

[14] Perez-Perez GI, Salomaa A, Kosunen TU, Daverman B, Rautelin H (2002). Evidence that cagA(+) Helicobacter pylori strains are disappearing more rapidly than cagA(−) strains.. Gut.

[15] Blaser MJ (2005). The biology of cag in the Helicobacter pylori-human interaction.. Gastroenterology.

[16] Odenbreit S, Puls J, Sedlmaier B, Gerland E, Fischer W (2000). Translocation of Helicobacter pylori CagA into gastric epithelial cells by type IV secretion.. Science.

[17] Suerbaum S, Michetti P (2002). Helicobacter pylori infection.. N Engl J Med.

[18] Kuipers EJ, Perez-Perez GI, Meuwissen SG, Blaser MJ (1995). Helicobacter pylori and atrophic gastritis: importance of the cagA status.. J Natl Cancer Inst.

[19] Everhart JE, Kruszon-Moran D, Perez-Perez G (2002). Reliability of Helicobacter pylori and CagA serological assays.. Clin Diagn Lab Immunol.

[20] Thjodleifsson B, Asbjornsdottir H, Sigurjonsdottir RB, Gislason D, Olafsson I (2007). Seroprevalence of Helicobacter pylori and cagA antibodies in Iceland, Estonia and Sweden.. Scand J Infect Dis.

[21] Chen Y, Blaser MJ (2007). Inverse associations of Helicobacter pylori with asthma and allergy.. Arch Intern Med.

[22] Chen Y, Blaser MJ (2008). Helicobacter pylori colonization is inversely associated with childhood asthma.. J Infect Dis.

[23] Burney PG, Laitinen LA, Perdrizet S, Huckauf H, Tattersfield AE (1989). Validity and repeatability of the IUATLD (1984) Bronchial Symptoms Questionnaire: an international comparison.. Eur Respir J.

[24] Asher MI, Keil U, Anderson HR, Beasley R, Crane J (1995). International Study of Asthma and Allergies in Childhood (ISAAC): rationale and methods.. Eur Respir J.

[25] Enright PL, McClelland RL, Newman AB, Gottlieb DJ, Lebowitz MD (1999). Underdiagnosis and undertreatment of asthma in the elderly. Cardiovascular Health Study Research Group.. Chest.

[26] Blaser MJ, Perez-Perez GI, Kleanthous H, Cover TL, Peek RM (1995). Infection with Helicobacter pylori strains possessing cagA is associated with an increased risk of developing adenocarcinoma of the stomach.. Cancer Res.

[27] Romero-Gallo J, Perez-Perez GI, Novick RP, Kamath P, Norbu T (2002). Responses of endoscopy patients in Ladakh, India, to Helicobacter pylori whole-cell and Cag A antigens.. Clin Diagn Lab Immunol.

[28] ATS (1995). Standardization of Spirometry, 1994 Update. American Thoracic Society.. Am J Respir Crit Care Med.

[29] Hankinson JL, Odencrantz JR, Fedan KB (1999). Spirometric reference values from a sample of the general U.S. population.. Am J Respir Crit Care Med.

[30] Hanley JA, Negassa A, Edwardes MD, Forrester JE (2003). Statistical analysis of correlated data using generalized estimating equations: an orientation.. Am J Epidemiol.

[31] Banatvala N, Mayo K, Megraud F, Jennings R, Deeks JJ (1993). The cohort effect and Helicobacter pylori.. J Infect Dis.

[32] Malaty HM, El-Kasabany A, Graham DY, Miller CC, Reddy SG (2002). Age at acquisition of Helicobacter pylori infection: a follow-up study from infancy to adulthood.. Lancet.

[33] Everhart JE, Kruszon-Moran D, Perez-Perez GI, Tralka TS, McQuillan G (2000). Seroprevalence and ethnic differences in Helicobacter pylori infection among adults in the United States.. J Infect Dis.

[34] Cassino C, Berger KI, Goldring RM, Norman RG, Kammerman S (2000). Duration of asthma and physiologic outcomes in elderly nonsmokers.. Am J Respir Crit Care Med.

[35] Sorkness RL, Bleecker ER, Busse WW, Calhoun WJ, Castro M (2008). Lung function in adults with stable but severe asthma: air trapping and incomplete reversal of obstruction with bronchodilation.. J Appl Physiol.

[36] Moore WC, Bleecker ER, Curran-Everett D, Erzurum SC, Ameredes BT (2007). Characterization of the severe asthma phenotype by the National Heart, Lung, and Blood Institute's Severe Asthma Research Program.. J Allergy Clin Immunol.

[37] Everhart JE (2000). Recent developments in the epidemiology of Helicobacter pylori.. Gastroenterol Clin North Am.

[38] Mai UE, Perez-Perez GI, Allen JB, Wahl SM, Blaser MJ (1992). Surface proteins from Helicobacter pylori exhibit chemotactic activity for human leukocytes and are present in gastric mucosa.. J Exp Med.

[39] O'Keeffe J, Moran AP (2008). Conventional, regulatory, and unconventional T cells in the immunologic response to Helicobacter pylori.. Helicobacter.

[40] Guiney DG, Hasegawa P, Cole SP (2003). Helicobacter pylori preferentially induces interleukin 12 (IL-12) rather than IL-6 or IL-10 in human dendritic cells.. Infect Immun.

[41] Bergman MP, Engering A, Smits HH, van Vliet SJ, van Bodegraven AA (2004). Helicobacter pylori modulates the T helper cell 1/T helper cell 2 balance through phase-variable interaction between lipopolysaccharide and DC-SIGN.. J Exp Med.

[42] Hafsi N, Voland P, Schwendy S, Rad R, Reindl W (2004). Human dendritic cells respond to Helicobacter pylori, promoting NK cell and Th1-effector responses in vitro.. J Immunol.

[43] Segal ED, Cha J, Lo J, Falkow S, Tompkins LS (1999). Altered states: involvement of phosphorylated CagA in the induction of host cellular growth changes by Helicobacter pylori.. Proc Natl Acad Sci U S A.

[44] Lundgren A, Stromberg E, Sjoling A, Lindholm C, Enarsson K (2005). Mucosal FOXP3-expressing CD4+ CD25high regulatory T cells in Helicobacter pylori-infected patients.. Infect Immun.

[45] Beswick EJ, Pinchuk IV, Das S, Powell DW, Reyes VE (2007). Expression of the programmed death ligand 1, B7-H1, on gastric epithelial cells after Helicobacter pylori exposure promotes development of CD4+ CD25+ FoxP3+ regulatory T cells.. Infect Immun.

[46] Raitala A, Karjalainen J, Oja SS, Kosunen TU, Hurme M (2005). Indoleamine 2,3-dioxygenase (IDO) activity is lower in atopic than in non-atopic individuals and is enhanced by environmental factors protecting from atopy.. Mol Immunol.

[47] Oderda G, Vivenza D, Rapa A, Boldorini R, Bonsignori I (2007). Increased interleukin-10 in Helicobacter pylori infection could be involved in the mechanism protecting from allergy.. J Pediatr Gastroenterol Nutr.

[48] Wilson MS, Taylor MD, Balic A, Finney CA, Lamb JR (2005). Suppression of allergic airway inflammation by helminth-induced regulatory T cells.. J Exp Med.

[49] Ishihara S, Rumi MA, Kadowaki Y, Ortega-Cava CF, Yuki T (2004). Essential role of MD-2 in TLR4-dependent signaling during Helicobacter pylori-associated gastritis.. J Immunol.

[50] Garantziotis S, Hollingsworth JW, Zaas AK, Schwartz DA (2008). The effect of toll-like receptors and toll-like receptor genetics in human disease.. Annu Rev Med.

[51] Harding SM, Richter JE (1997). The role of gastroesophageal reflux in chronic cough and asthma.. Chest.

[52] Kiljander TO, Laitinen JO (2004). The prevalence of gastroesophageal reflux disease in adult asthmatics.. Chest.

[53] Harding SM (2005). Gastroesophageal reflux: a potential asthma trigger.. Immunol Allergy Clin North Am.

[54] Peek RM, Blaser MJ (2002). Helicobacter pylori and gastrointestinal tract adenocarcinomas.. Nat Rev Cancer.

